# Editorial: Multidisciplinary management of oral cancer: diagnosis, treatment, and rehabilitation

**DOI:** 10.3389/froh.2025.1747684

**Published:** 2025-12-08

**Authors:** Oreste Iocca, Camila Lopes Cardoso, Pasquale Di Maio

**Affiliations:** 1 Division of Maxillofacial Surgery, Città Della Salute e Della Scienza Hospital, University of Torino, Torino, Italy; 2 Doctoral Degree in Health Sciences - Grupo de Terapia Celular Y Medicina Regenerativa, Departamento de Fisioterapia, Medicina Y Ciencias Biomédicas, Facultad de Ciencias de la Salud, Universidade da Coruña, A Coruña, Spain; 3 Department of Surgery, Stomatology, Pathology and Radiology, Bauru School of Dentistry, University of São Paulo, São Paulo, Brazil; 4 Doctoral Degree in Translational Research in Public Health and High Prevalence Diseases, UIB - Universitat de les Illes Balears, Palma de Mallorca, Spain; 5 Department of Otolaryngology-Head and Neck Surgery, Giuseppe Fornaroli Hospital, ASST Ovest Milanese, Milan, Italy

**Keywords:** head and neck cancer, oral cancer, squamous cell carcinoma, microvascular reconstruction, multidiscipinary

Head and neck cancer (HNC) comprise a group of malignancies that originate from various anatomical subsites, including the oral cavity, oropharynx, nasopharynx, paranasal sinuses, hypopharynx, larynx, and salivary glands. The predominant histologic type is squamous cell carcinoma (1), which is typically aggressive and challenging to treat with curative intent. The most common etiologic factors are smoking and alcohol consumption, while human papilloma virus (HPV) infection has a role especially in the etiology of oropharynx carcinoma (2). HNC most commonly spread to regional lymph nodes of the neck, with distant metastases to other organs via hematogenous routes occurring less frequently (3, 4) Treatment options consist of surgery, radiotherapy, and chemotherapy, which are typically used in combination or in sequence. Immunotherapy has emerged as a potential treatment option in both the adjuvant or neoadjuvant setting (5).

Oral cavity carcinoma (OCC) represents a significant global public health problem (6). According to the most recent estimates available from the global cancer observatory (7), it is the 16th most common cancer worldwide with an estimated incidence of 389'846 cases and 188'438 deaths. Management of OCC is complex and a careful interplay of surgery, radiotherapy, and chemotherapy guarantees higher chances of survival and the best quality of life (QoL) after treatment. Also, perioperative care and treatment decisions must be taken in accordance with the best available scientific evidence, clinicians experience, and patients' preferences. For this reason, the focus of this research topic was on the multidisciplinary management of this elusive disease.

Every new oral cancer diagnosis should be discussed on a multidisciplinary tumor board (MTB), this is important in order to take appropriate therapeutic decisions and expedite the treatment pathways. The MTB discussion should also serve as a platform where surgeons, pathologists, radiologists, radiotherapists, and supportive care specialists can discuss areas of uncertainty, whether related to diagnostic interpretation or optimal treatment strategy. The paper by Burkhardt et al. evaluated the potential benefits of pretherapeutic MTB for OCC. The study analyzed data from a group of patients which received no pretherapeutic presentation and a group which received pretherapeutic MTB discussion. The results showed that MTB presentation led to a higher adherence to national guidelines compared to the non-MTB group. Also, a tendency toward a higher 5-year survival was shown, although this was not statistically significant. On the other hand, the MTB group showed a higher diagnosis-to-treatment delay. The authors suggest that this observed delay in treatment initiation may be offset by improved coherence with the guideline. These results confirm the importance of MTB discussion in providing the best evidence-based care to OCC patients and, potentially, increasing the survival chances.

Another aspect to consider to improve the treatment outcomes of OCC is a proper and timely diagnosis. The current clinical standard for diagnosis is clinical inspection followed by biopsy and histopathologic confirmations. Recently, the potential use of biomarkers has emerged as a future diagnostic tool. The paper by Liu et al. reviewed the advancements in OCC biomarker detection and their potential future clinical implementation. Their summary focused on sequencing technologies, biosensors, artificial intelligence (AI), and other techniques. They conclude that biomarker detection is poised to revolutionize clinical practice, although high cost, inconsistencies in data interpretation, and lack of standardized validation frameworks are barriers that need to be overcome for the adoption of these technologies in everyday clinical practice. The paper of Abudukelimu et al. investigated the role of laboratory values as an additional aid in the diagnosis of OCC, including Carbohydrate antigen 125 (CA125), neuron-specific enolase (NSE), neutrophil-to-lymphocyte ratio (NLR), platelet-to-lymphocyte ratio (PLR), and systemic immune-inflammatory response index (SIRI). They showed that NLR, PLR, and SIRI showed significant correlations with primary tumor size and local lymph node involvement. Moreover, NLR and SIRI exhibited strong associations with distant metastasis. They concluded that the altered values of specific laboratory biomarkers such as CA125, NSE, NLR, PLR, SIRI, may be associated with tumor onset and progression. These results, if validated by larger prospective clinical studies, opens future possibilities of obtaining more accurate diagnoses and more precise pretreatment therapeutic decisions. Another laboratory marker was evaluated by Maturana-Ramirez et al. who presented a study correlating hypovitaminosis D with the presence of oral leukoplakia (OL), an oral potentially malignant disorder known to transform in OCC in around 9.5% of the cases (8). The study demonstrated that serum vitamin D levels were lower in patients with OL compared to those without OL, with no differences related to histopathological diagnosis. This finding is consistent with previous research and suggests a potential role for vitamin D deficiency in the development of OL, with tobacco possibly acting as a synergistic agent. These results suggest the potential for further research into the use of vitamin D as a chemopreventive agent in the malignant transformation of OL.

The perioperative care of patients has emerged over the last two decades as a fundamental factor influencing the survival outcomes and the QoL of patients affected by head and neck cancer. The research by Kuang et al. focused on preoperative evaluation to predict the occurrence of postoperative malnutrition in OCC patients. They used various machine learning algorithms using the Shapley Additive exPlanations (SHAP) for their model explanation. The results showed that the model extreme gradient boosting (XGBoost) significantly predicted seven key predictors of post-operative malnutrition including sex, T stage, repair and reconstruction, diabetes status, age, lymphocyte count, and total cholesterol level. Further validation of these results will help the multidisciplinary team in implementing perioperative treatment strategies for the prevention of post-operative malnutrition. Another important aspect to consider in the perioperative setting is the QoL after treatment, Li et al. evaluated the impact of cervical osteoarthritis on QoL after free flap reconstruction in head and neck cancer. The authors illustrate that cervical osteoarthritis correlates with transient postoperative functional impairments and persistent fatigue but does not adversely affect long-term QoL. These findings outline the importance of preoperative counseling, post-operative rehabilitation measures, and routine screening for fatigue after completion of therapy. An interesting result emerging from this paper was the observed emotional resilience in cervical osteoarthritis patients which was evident as heightened coping mechanisms. Patterson et al. evaluated the feasibility and acceptability of integrating a remote, personalized, collaborative, and flexible exercise program into the head and neck cancer care pathway. They included patients between diagnosis and 8 weeks post-treatment evaluating retention and exercise adherence were primary outcomes. Quantitative outcomes included quality-of-life, fatigue and physical activity questionnaires and physical fitness tests. The results showed that around half of the included patients completed the intervention. Overall, patients valued positively the program, for both their physical and mental well-being. This further confirms that a holistic approach to patient care must be integrated in all multidisciplinary teams treating head and neck cancer. The study by Hoene et al. focused on an important aspect of doctor-patient relationship, namely communication. The authors proved that wearing face masks does not significantly impair perceived empathy in the context of physician-patient communication. This finding suggests that implementing a simple yet important safety measure for both healthcare personnel and patients does not compromise the quality of care.

Regarding OCC treatment, surgery is the first treatment option with microvascular reconstruction often needed after tumor ablation, usually followed by radiotherapy or chemoradiotherapy according to specific disease characteristics. Access to the best specialized care if thus fundamental in order to obtain the best chances of survival. The paper by Goodnight et al. examined geospatial analysis from various public databases in the United States. It was shown that significant regional disparities in access to head and neck specialists exist, with non-metropolitan areas and regions outside the Northeast showing notably longer travel times. Socioeconomic and demographics factors, including lower household income, lower insurance coverage, and higher median age, were associated with increased travel times. The disparity index developed by the study could serve as a tool for identifying high-need areas and guiding policy interventions. This kind of research is useful to understand how to best allocate resources, implement mobile clinics, and create incentives for improving access to specialized care.

Microvascular reconstruction provides the best treatment results after ablation although this type of surgery has an unavoidable impact on the QoL. Zhao et al. examined the functional outcomes and quality of life following free fibula flap (FFF) harvest with either flexor hallucis longus (FHL) resection or its preservation. The results showed that resecting the FHL during FFF harvest produced only temporary functional and quality-of-life impairments, most of which were resolved within six months. The authors concluded that preservation of the FHL is recommended when feasible. On the other hand, its harvest remains a safe option when additional soft tissue is required. Fenske et al. studied the occurrence of osteoradionecrosis (ORN) in osseous free flaps after maxillofacial reconstruction. They concluded that osseous free flap ORN is a severe complication which often leads to total flap loss especially in patients that smoke and those who had prior reconstruction plate exposure. This outlines the importance of careful preoperative evaluation when performing reconstruction with an osseous flap in patients in which radiotherapy will be likely indicated.

Neck dissection is often indicated in patients affected by OCC (9). Sentinel lymph node biopsy (SNLB) is not widely adopted but is performed in some specialized centers. Wang et al. examined whether sentinel lymph node biopsy provides better regional control than observation in early stage maxillary squamous cell carcinoma. Results demonstrated superior regional control of SNLB patients compared to observation in patients diagnosed with cT1/2N0 maxillary OCC. Future studies will be needed to compare elective neck dissection vs. SNLB. Wei et al. explored the effectiveness of ultrasound-guided microwave ablation combined with radiotherapy and chemotherapy for patients with poor performance status or those unwilling to undergo surgery. In this context, encouraging results have been demonstrated concerning the safety and minimally invasive nature of treatment for advanced tongue cancer.

In conclusion, the valuable papers published in this Research Topic show that the research field regarding OCC management is dynamic and rapidly evolving, offering exciting opportunities for future developments and innovations. Once again, it has been shown that multidisciplinary efforts are fundamental to obtaining optimal care for patients afflicted by OCC. Future developments in individualized care and more refined treatment modalities will guarantee improved survival outcomes and better quality of life results.
